# A Potential Endurance Algorithm Prediction in the Field of Sports Performance

**DOI:** 10.3389/fgene.2020.00711

**Published:** 2020-08-11

**Authors:** Rocio de la Iglesia, Isabel Espinosa-Salinas, F. Javier Lopez-Silvarrey, J. Jose Ramos-Alvarez, J. Carlos Segovia, Gonzalo Colmenarejo, Elena Borregon-Rivilla, Helena Marcos-Pasero, Elena Aguilar-Aguilar, Viviana Loria-Kohen, Guillermo Reglero, Ana Ramirez-de Molina

**Affiliations:** ^1^Departamento de Ciencias Farmacéuticas y de la Salud, Facultad de Farmacia, Universidad San Pablo-CEU, CEU Universities, Alcorcón, Spain; ^2^Nutrition and Clinical Trials Unit, GENYAL Platform IMDEA-Food Institute, CEI UAM + CSIC, Madrid, Spain; ^3^Facultad de Ciencias de la Salud, Universidad Camilo José Cela, Madrid, Spain; ^4^Sannus Clinic, Madrid, Spain; ^5^Departamento de Radiología, Rehabilitación y Fisioterapia, Universidad Complutense de Madrid, Madrid, Spain; ^6^Biostatistics and Bioinformatics Unit, IMDEA Food CEI UAM + CSIC, Madrid, Spain; ^7^Department of Production and Characterization of Novel Foods, Institute of Food Science Research (CIAL) CEI UAM + CSIC, Madrid, Spain

**Keywords:** SNP, genetics, exercise, functional validation, nutrition

## Abstract

Sport performance is influenced by several factors, including genetic susceptibility. In the past years, specific single nucleotide polymorphisms have been associated to sport performance; however, these effects should be considered in multivariable prediction systems since they are related to a polygenic inheritance. The aim of this study was to design a genetic endurance prediction score (GES) of endurance performance and analyze its association with anthropometric, nutritional and sport efficiency variables in a cross-sectional study within fifteen male cyclists. A statistically significant positive relationship between GES and the VO_2_ maximum (*P* = 0.033), VO_2_ VT1 (*P* = 0.049) and VO_2_ VT2 (*P* < 0.001) was observed. Moreover, additional remarkable associations between genotype and the anthropometric, nutritional and sport performance variables, were achieved. In addition, an interesting link between the habit of consuming caffeinated beverages and the GES was observed. The outcomes of the present study indicate a potential use of this genetic prediction algorithm in the sports’ field, which may facilitate the finding of genetically talented athletes, improve their training and food habits, as well as help in the improvement of physical conditions of amateurs.

## Introduction

Athletic performance can be influenced by several factors. These can include extrinsic factors such as the quality of training, the dietary habits, the technology used or the weather conditions (Sacha and Quinn, 2011). Besides, intrinsic factors such as individual genetic profile also play an important role (Peveler and Green, 2010). In fact, the study of the genetic influence on sports performance has become a leading field of research.

Single nucleotide polymorphisms (SNPs) are among the genetic variants implicated in the phenotypic differences that can influence individual physical abilities (Santos et al., 2016). To date, various links between several genetic variants and favorable phenotypes for certain sports have been established (Santos et al., 2016). This suggests that the presence of specific genotypes can predispose an individual to competitive advantages in a particular sport (Zilberman-Schapira et al., 2012).

For example, backed by a meta-analysis, the II genotype of the angiotensin converting enzyme *ACE* insertion/deletion (I/D) rs4340 has been significantly associated with endurance’s discipline as compared with the ID and DD genotypes (Ma et al., 2013). Specifically, it is suggested that the I allele implies higher blood flow and sugar utilization by muscles, that facilitates endurance performance (Woods et al., 2000). Additionally, considering reported difficulties in genotyping *ACE* I/D, the *ACE* rs4343 A and G alleles are admitted as equivalents to the *ACE* rs4340 I and D alleles, respectively, and considered an alternative method for genotyping of the *ACE* I/D polymorphism (Glenn et al., 2009).

Moreover, the different genotypes of the peroxisome proliferator activated receptor alpha PPARA rs4253778 have also been associated with athlete’s performance. Concretely, in a meta-analysis carried out by Lopez-Leon et al. (2016) the GG genotype and G allele were significantly more common in endurance athletes as compared to controls.

Besides, the α-actinin 3 (ACTN3) is a sarcomeric actin-binding protein specifically expressed in fast twitch myofibers of the skeletal muscle, required for explosive muscle contraction (Wilson et al., 2012). But the gene encoding this protein also seems to be associated with endurance capacities. In particular, TT rs1815739 carriers were found to be more common in endurance athletes as compared with sedentary individuals in a sample of 395 Israeli (Eynon et al., 2009). They were also described to obtain better results in an endurance test (Pasqua et al., 2016), to exhibit a higher proportion of endurance-associated type I myofibers and to prefer to skate long- than short-distance races (Ahmetov et al., 2011).

Another gene with a potential association with endurance capacities is the Aquaporin 1 *AQP1* rs1049305 where C allele carriers were faster in running performances than carriers of the GG genotype (Martinez et al., 2009; Rivera et al., 2011; Saunders et al., 2015).

Moreover, the CC genotype of the peroxisome proliferator activated receptor gamma, coactivator 1 alpha *PPARGC1A* rs8192678 has been described to be associated with high values of aerobic performance (Akhmetov et al., 2007; Stefan et al., 2007; Eynon et al., 2010b).

And finally, other SNPs such as the beta-3 adrenergic receptor *ADRB3* rs4994 (Santiago et al., 2011), the GA-binding protein transcription factor, subunit beta 1 *GABPB1* rs12594956 (Eynon et al., 2010a, 2013), the collagen type V alpha 1 chain *COL5A1* rs12722 (Brown et al., 2011; Posthumus et al., 2011) and the hemochromatosis *HFE* rs1799945 (Grealy et al., 2015) have also been associated with better endurance performance, although the scientific evidence support for these associations is still scarce.

Thus, it is clear that there is a genetic influence, but there is still a weak scientific evidence for most of the reported associations. Besides, most studies that associate genetics with physical capacities have focused on individual genes. However, as a polygenic inheritance, various genes can make a contribution to the overall outcome. For these reasons, we consider that it is necessary to create more complex prediction algorithms, including different genetic factors together. Therefore, in the present study, we have developed a predictive algorithm of endurance performance including 11 genes. In this report, we present the associations of the predictive algorithm and each of the SNPs with anthropometric, nutritional and sport performance variables, in a group of 15 semi-professional cyclists.

## Materials and Methods

### Subjects

Fifteen healthy male members of the Spanish Cycling Federation, body mass index (BMI) 22.3 ± 2.5 aged 40.7 ± 7.0, with at least 5 years of experience in national-level competitions were recruited by the Sports Medicine University Center (Complutense University of Madrid, Madrid, Spain) to participate in the present study. All participants were non-smokers. This research was conducted according to the guidelines laid down in the Declaration of Helsinki and all procedures involving human subjects were approved by the Research Ethics Committee of the IMDEA Food Foundation (PI-0031). Written informed consent to participate in the study was obtained from all subjects.

### Design

This was a cross-sectional clinical study where volunteers attended two different centers to complete the study:

1) The Sports Medicine University Center to carry out a maximal incremental treadmill test.
2) The Research Institute on Food and Health Sciences “IMDEA Food” (Madrid, Spain) for anthropometric measurements, body composition analysis, dietary records, DNA collection and genotyping.

### Methodology

#### Cardiopulmonary Exercise Test

All participants carried out an incremental (30 w/min) exercise testing on a cycle ergometer (Cardgirus, Barcelona, Spain) after 2 h of fasting. During the test, the heart rate (HR) was measured using a 12-lead wireless electrocardiograph (Norav, Wiesbaden, Germany).

Oxygen uptake (VO_2_), carbon dioxide output (VCO_2_) and minute ventilation (VE) were assessed using the analyzer Jaeger Oxycon-Pro (Hoechberg, Germany). The respiratory exchange ratio (RER) was calculated as VCO_2_/VO_2_, while the VO_2_ pulse as VO_2_/HR (Bergh et al., 2000). Every 2 min cyclists had to estimate their feelings of exertion and pain using the Borg Rating of Perceived Exertion (RPE) Scale (Borg et al., 1985).

All participants achieved maximal exercise criteria: VO_2_ plateau (considered as VO_2_ max) which was estimated as a VO_2_ increase lower than 150 mL/min for two consecutive periods (Petot et al., 2012), RPE higher than 16 (Bergh et al., 2000), RER above 1.10 and a HR upper 90% of the theoretical maximum HR (Howley et al., 1995). The maximum HR was calculated as [(208.75−(0.73 × age)] (Tanaka et al., 2001).

The aerobic ventilatory threshold (VT1) was estimated using the criteria of the ventilatory equivalent for VO_2_ (VE/VO_2_ ratio), corresponding to the rupture of the linearity in the increment of VE. Finally, the anaerobic ventilatory threshold (VT2) was calculated by the increase of the VCO_2_ equivalent principles (VE/VCO_2_), as the second rupture of the linearity in the increment of VE (Beaver et al., 1986).

#### Anthropometry and Lifestyle Parameters

Anthropometric measurements were determined while subjects were wearing light clothing and no shoes. Height was assessed to the nearest 0.1 cm using a stadiometer (Leicester-Biological Medical Technology SL, Barcelona). Body weight, fat mass and muscle mass percentages were evaluated using a BF511 Body Composition Monitor (BF511- OMRON Healthcare UK, LT, Kyoto, Japan). Brachial, contracted arm, waist, hip and leg circumferences were measured with an inextensible tape (KaWe Kirchner & Wilhelm GmbH, Asperg, Germany; range 0–150 cm, 1 mm of precision). A caliper (Holtain Ltd., Crymych, United Kingdom; 10 g/mm^2^ constant pressure; range 0–39 mm and 0.1 mm of precision) was used for biceps, triceps, subscapular, abdominal, supraspinal, front thigh and medial calf skinfolds determinations. Moreover, the diameters of the femur and humerus were also assessed using a small bone caliper (Nonio sliding Bicondyleo, Holtain Ltd., United Kingdom). Systolic and diastolic blood pressures were evaluated using an automatic digital blood pressure monitor Model M3 (OMRON Healthcare UK, LT, Kyoto, Japan) in the right arm, with the patient seated and relaxed. Measurements were taken three times after a 5-min resting period, following World Health Organization (WHO) criteria (Whitworth and Chalmers, 2004). Finally, with the different anthropometric data, the BMI was calculated as the body weight divided by the squared height (kg/m^2^) and somatotype values (endomorphic, mesomorphic, and ectomorphic values) accordingly to Heath-Carter method (Carter and Heath, 1990).

The food habits of each participant were recorded using a validated 3-day dietary food record and a food frequency questionnaire (Aguirre-Jaime et al., 2008). Subsequently, the composition of the different dietary records was analyzed using the DIAL software (2.16 version Alce Ingeniería, Madrid, Spain). For the calculation of the Healthy Eating Index score (Guenther et al., 2013), the DIAL program gives different values ranging from 0 to 100 considering the daily servings of cereals, vegetables, fruits, dairy products, and meat; the percentage of energy provided by total and saturated fats; the amount of cholesterol and sodium per day and the number of different foods consumed. The final score is classified into five categories: an “excellent diet” (>80 points), a “very good diet” (71–80 points), a “good diet” (61–70 points), an “acceptable diet” (51–60 points), or an “inadequate diet” (0–50 points).

#### Genotyping of the Population

A sample of 500 μl of peripheral capillary blood of each volunteer was drawn for DNA extraction. To perform the subsequent analysis of the samples, genomic DNA was extracted from the cellular fraction collected by the Genomic QIAamp DNA Blood Kit Mini Kit (QIAGEN, Spain). The samples were genotyped with TaqMan Assays by the high-performance QuantStudio Real-Time PCR (Applied Biosystem, United States).

### Statistical Analysis

Data were analyzed using the R Statistical Software Version 3.4.1^1^. The description of the qualitative data was made in the form of absolute frequencies and percentages and the quantitative data by mean and standard deviation. The Mann–Whitney U test was used to check for significant differences in the continuous variables (not always normally distributed) for the different genotypes. The Spearman correlation coefficient was used for the association between the algorithm and the other variables. The Bonferroni correction was also applied to control against type-I errors for multiple tests. All the statistical tests were two-tailed. Statistical significance was assumed when *P* < 0.05.

### Selection of SNPs and Design of the GES

An exhaustive literature review of the scientific databases (Pubmed, Medline, Web of Sciences) was carried out to identify all studies that analyzed the relationship between one or more SNP and sports performance. A selection of 11 SNPs was made considering the European frequencies of each SNP according to Ensembl database, the scientific evidence of each association and its availability for TaqMan SNP Genotyping Assay. Among them, nine SNPs were associated with endurance capacities and two SNPs were mainly related to power abilities.

Once the SNPs selection was completed, an algorithm to predict endurance capacities based on the nine SNPs associated with endurance performance was established (Table 1). Depending on the scientific evidence, each SNP was given a different weight to the total GES. Therefore, the two SNPs with the highest scientific evidence based on meta-analysis, *ACE* and *PPAR*α, were given the highest normalized weight of the GES, contributing 22.2% each of the total value of the algorithm. On the other hand, those SNPs whose evidence was based on at least three studies with positive and conclusive results (*ACTN3*, *AQP1*, and *PPARGC1A*) were given a weight of 11.1% each, of the total value of the algorithm. Besides, to the remaining 4 SNPs, with less scientific evidence and some contradictory results, each one was given a weight of 5.6% to the final GES. Finally, based on the literature, the three possible genotypes of each SNP were classified into three categories: “favorable genotypes” (GES weight × 1), which were those associated with better endurance performance; “intermediate genotypes” (GES weight × 0.5), which were those with a neutral effect on endurance abilities, and “unfavorable genotypes” (GES weight × 0), which were those with a negative effect. For example, an individual with all favorable SNPs, would have a GES value of 100: 22.2 × 1 (*ACE*) + 22.2 × 1 (*PPAR*α) + 11.1 × 1 (*ACTN3*) + 11.1 × 1 (*AQP1*) + 11.1 × 1 (*PPARGC1A*) + 5.6 × 1 (*ADRB3*) + 5.6 × 1 (*GABPB1*) + 5.6 × 1 (*COL5A1*) + 5.6 × 1 (*HFE*) = 100. However, an individual with unfavorable *ACE* and *PPAR*α SNPs and all the other SNPs favorable, would have a score of 56: 22.2 × 0 (*ACE*) + 22.2 × 0 (*PPAR*α) + 11.1 × 1 (*ACTN3*) + 11.1 × 1 (*AQP1*) + 11.1 × 1 (*PPARGC1A*) + 5.6 × 1 (*ADRB3*) + 5.6 × 1 (*GABPB1*) + 5.6 × 1 (*COL5A1*) + 5.6 × 1 (*HFE*) = 56.

**TABLE 1:** Genes included in the genetic endurance prediction score (GES).

Gene (Complete name)	Gene (Acronym) SNP	Specific association	Genotypes classification	Frequency (%)	Contribution (%)	References
Angiotensin converting enzyme	*ACE* rs4343	Blood pressure regulation	AA favorable	19.1	22.2	Metanalysis (Ma et al., 2013)
			AG intermediate	48.9		
			GG unfavorable	32.0		
Alpha-actinin-3	*ACTN3* rs1815739	Muscle contraction	TT favorable	17.9	11.1	Eynon et al., 2009; Ahmetov et al., 2011; Pasqua et al., 2016
			CT intermediate	51.1		
			CC intermediate	31.0		
Beta-3 adrenergic receptor	*ADRB3* rs4994	Lipolysis and thermogenesis stimulation	GG favorable	0.8	5.6	Santiago et al., 2011
			AG favorable	14.7		
			AA unfavorable	84.5		
Aquaporin 1	*AQP1* rs1049305	Osmotic balance by water transport	CC favorable	15.5	11.1	Martinez et al., 2009; Rivera et al., 2011; Saunders et al., 2015
			CG favorable	46.9		
			GG unfavorable	37.6		
GA-binding protein transcription factor, β subunit 1	*GABPB1* rs12594956	Energy synthesis in mitochondria	AA favorable	38.0	5.6	Eynon et al., 2010a, 2013
			AC intermediate	44.9		
			CC intermediate	17.1		
Collagen type V alpha 1 chain	*COL5A1* rs12722	Fibrillogenesis in ligaments and tendons	TT favorable	35.6	5.6	Brown et al., 2011; Posthumus et al., 2011
			CT intermediate	45.9		
			CC intermediate	18.5		
Hemochromatosis	*HFE rs1799945*	Iron absorption	CG favorable	27.2	5.6	Grealy et al., 2015
			GG intermediate	3.6		
			CC intermediate	69.2		
Peroxisome proliferator activated receptor alpha	*PPAR*α *rs4253778*	Metabolism of energy, lipids and carbohydrates	GG favorable	65.6	22.2	Metanalysis (Lopez-Leon et al., 2016)
			CG intermediate	30.4		
			CC unfavorable	4.0		
Peroxisome proliferator activated receptor, gamma, coactivator 1, alpha	*PPARGC1A rs8192678*	Glucose transportation and lipid and glucose oxidation	CC favorable	41.2	11.1	Akhmetov et al., 2007; Stefan et al., 2007; Eynon et al., 2010b
			CT unfavorable	45.5		
			TT unfavorable	13.3		

*Genetic endurance prediction score (GES): 2.22 ACE + 2.22 PPARα + 1.11 ACTN3 + 1.11 AQP1 + 1.11 PPARGC1A + 5.6 ADRB3 + 5.6 GABPB1 + 5.6 COL5A1 + 5.6 HFE.*

The selected power-related genotypes were:

• Hypoxia inducible factor 1 alpha subunit *HIF1A* rs11549465: CC (81.1%) unfavorable, CT (17.7%) intermediate, TT (1.2%) favorable.
• Muscle-specific creatine kinase *CKM* rs8111989: TT (48.3%) unfavorable, CT (43.3%) intermediate, CC (8.3%) favorable.

## Results

### Descriptive Analysis

Mean values of anthropometric, body composition, somatotype and blood pressure of all cyclists are shown in Table 2. As expected, the mean BMI and fat mass percentage values were in the range of “normal weight” [18.5–24.9 BMI, 12–20% fat mass percentage according to the Spanish Society for the Study of Obesity (Salas-Salvado et al., 2007)]. The three somatotype components were around the moderate 3–5 rate (Carter and Heath, 1990).

**TABLE 2:** Anthropometric, body composition, somatotype and blood pressure variables.

Variables	Mean (SD)
Age (years)	40.67(6.97)
Body weight (kg)	70.49(8.09)
Height (cm)	177.99(4.71)
Body mass index	22.27(2.47)
Fat mass (%)	14.43(5.27)
Muscle mass (%)	40.61(3.63)
Visceral fat classification	4.43(2.59)
Waist circumference (cm)	78.95(5.28)
Waist to hip ratio	0.82(0.04)
Relaxed arm circumference (cm)	29.73(2.23)
Flexed and tensed arm circumference (cm)	31.58(2.21)
Biepicondylar humerus diameter (cm)	7.25(0.29)
Biepicondylar femur diameter (cm)	9.92(0.40)
Biceps skinfold (mm)	3.43(0.90)
Triceps skinfold (mm)	7.55(4.09)
Subscapular skinfold (mm)	9.05(2.36)
Supraspinal skinfold (mm)	9.4(6.03)
Abdominal skinfold (mm)	14.61(9.24)
Front thigh skinfold (mm)	9.42(4.98)
Calf circumference (cm)	36.89(2.08)
Medial calf skinfold (mm)	4.98(1.81)
Systolic blood pressure (mmHg)	126.8(7.04)
Diastolic blood pressure (mmHg)	76.4(6.38)
Endomorphic value	3.98(1.24)
Mesomorphic value	2.79(1.07)
Ectomorphic value	3.06(1.03)

Moreover, the results of the analysis of the 3-day dietary records can be observed in Table 3. Here, it can be appreciated how the energy requirements of the cyclists were slightly higher than the energy intakes, these lasts with a high variability between the participants. Moreover, the mean Healthy Eating Index resulted in a “good diet” which, as explained in the methodology, is considered an intermediate value (61–70 points). This table also shows the average of servings per group of food consumed according to the Validated Food Frequency Questionnaire, where we can observe that vegetables and fruits are the most consumed.

**TABLE 3:** Dietary data.

Variables	Mean (SD)	Variables	Mean (SD)
Energy requirements (kcal)	3370(348)	Energy intake (kcal)	3192(1123)
Proteins (% TCV)	16.35(3.67)	Carbohydrates (% TCV)	43.5(10.62)
Sugars (% TCV)	19.62(7.40)	Lipids (% TCV)	36.91(9.22)
Saturated fatty acids (% TCV)	11.39(3.31)	Healthy Eating Index	65.19(14.06)
Glycemic index	48.96(11.58)	Vegetables (s/d)	2.66(1.32)
Fish and seafood (s/d)	0.92(0.56)	Fruits (s/d)	2.98(2.10)
Legumes (s/d)	0.39(0.28)	Nuts (s/d)	0.68(1.26)
Dairy (s/d)	2.53(1.20)	Eggs (s/d)	0.53(0.25)
Red meat (s/d)	0.45(0.21)	White meat (s/d)	0.48(0.21)
Processed meat (s/d)	1.09(0.94)	Animal fats (s/d)	0.26(0.40)
Viscera (s/d)	0.02(0.04)	Ready to eat foods (s/d)	0.19(0.13)
Salty snacks (s/d)	0.38(0.34)	Sauces (s/d)	0.23(0.15)
Alcohol (s/d)	0.32(0.28)	Coffee and tea (s/d)	1.28(1.33)
Garlic and spices (s/d)	0.57(0.52)	Pastries and sweets (s/d)	2.00(0.98)

*s/d, servings per day; TCV, total caloric value.*

Finally, Table 4 describes the mean results of the cardiopulmonary exercise test carried out at the Sports Medicine University Center.

**TABLE 4:** Cardiopulmonary exercise test output.

Variables	Mean (SD)
Maximal heart rate (bpm)	176.20 (15.23)
Resting heart rate (bpm)	51.40 (7.03)
Heart rate in VT1 (bpm)	131.67 (15.25)
Heart rate in VT2 (bpm)	157.67 (14.07)
VO_2_ max (mL/min)	4116 (565)
VO_2_ max (mL/kg/min)	59.16 (6.50)
VO_2_ in VT1 (mL/kg/min)	39.59 (8.08)
VO_2_ in VT2 (mL/kg/min)	50.61 (6.19)
VCO_2_ max (mL/min)	5136 (777)
VCO_2_ max (mL/kg/min)	73.85 (10.41)
Maximum minute ventilation (L/min)	169.80 (23.99)
Minute ventilation in VT1 (L/min)	58.21 (13.78)
% VO_2_ in VT1 in relation to VO_2_ max	66.94 (9.13)
% VO_2_ in VT2 in relation to VO_2_ max	85.95 (5.53)
Minute ventilation in VT2 (L/min)	93.07 (16.13)
O_2_ pulse max	23.53 (3.36)
O_2_ pulse in VT1	20.87 (3.28)
O_2_ pulse in VT2	22.47 (3.41)
Highest workload achieved	329.00 (42.74)
Workload in VT1	186.27 (35.20)
Workload in VT2	261.67 (38.94)

*VCO_2_ max, maximal carbon dioxide output; O_2_ pulse, VO_2_/heart rate; VO_2_ max, maximal oxygen consumption; VT1, aerobic ventilatory threshold; VT2, anaerobic ventilatory threshold.*

### Association Analyses

#### The GES Correlates With the Cardiovascular Exercise Test

**FIGURE 1:**
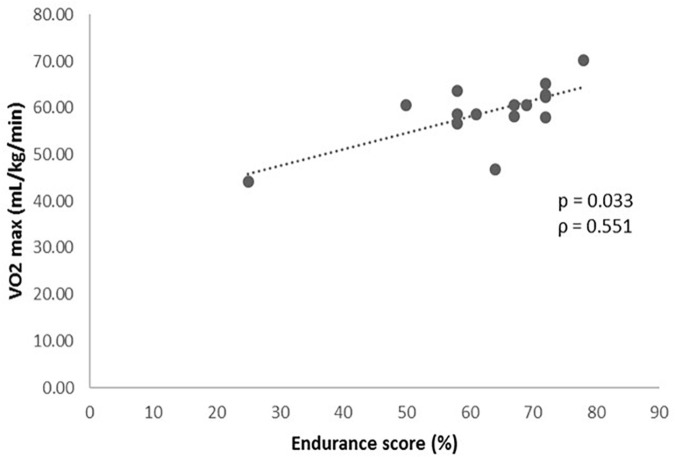
Levels of VO_2_ max (mL/kg/min) during the cardiopulmonary exercise test according to the genetic endurance prediction score (GES).

Once established the prediction algorithm (Table 1), it was related to the results obtained from the functional cardiovascular exercise test (Table 2). The association analysis of the GES and the variables obtained in the cardiovascular exercise test, revealed a statistically significant positive relationship between the GES results and the VO_2_ max (*P* = 0.033, Figure 1), VO_2_ in VT1 (*P* = 0.049, ρ = 0.516) and VO_2_ in VT2 (*P* < 0.001, ρ = 0.813) values.

**FIGURE 2:**
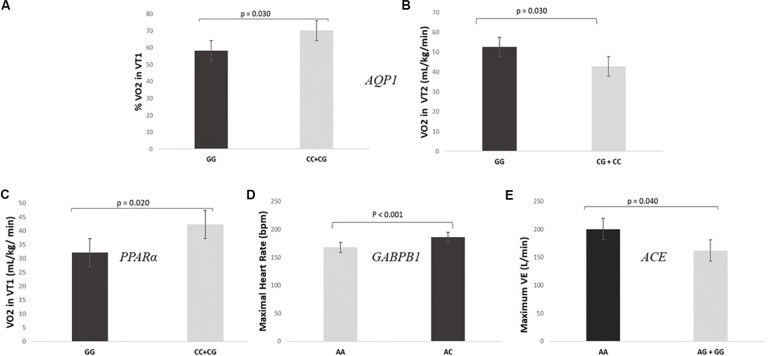
SNPs included in the prediction algorithm GES individually associated to specific parameters related to individual performance. **(A,B)** Levels of VO_2_ and% VO_2_ in VT1 during the cardiopulmonary exercise test according to *AQP1* rs1049305. **(C)** Levels of VO_2_ in VT2 during the cardiopulmonary exercise test according to *PPAR*α rs4253778. **(D)** Maximal heart rate during the cardiopulmonary exercise test according *GABPB1* rs12594956. **(E)** Maximum minute ventilation (VE) during the cardiopulmonary exercise test according *ACE* rs4343.

As expected, SNPs included in the GES were also individually associated with specific parameters related to individual performance. The analysis of the association of the different genotypes individually and the cardiopulmonary exercise test showed that GG genotypes for *AQP1* showed lower levels of VO_2_ in VT1 (32.1 ± 4.6 mL/kg/min vs 42.3 ± 7.4 mL/kg/min) and fewer values of% VO_2_ in VT1 in relation to VO_2_ max (58.2 ± 7.0% vs 70.1 ± 7.7%) than CC + CG (*P* = 0.020 and *P* = 0.030, respectively) (Figures 2A,B). On the other hand, GG genotypes for the *PPAR*α presented higher values of VO_2_ in VT2 than CC + CG (52.6 ± 4.6 mL/kg/min vs 42.8 ± 5.9 mL/kg/min, *P* = 0.030), as shown in Figure 2C.

Regarding the *GABPB1*, AA genotypes had significantly lower maximal HR (Figure 2D) and minor HR in VT1 and VT2 than AC genotypes (167.8 ± 16.5 bpm vs 185.9 ± 4.5 bpm, 120.8 ± 11.0 bpm vs 144.1 ± 7.8 bpm and 149.0 ± 13.3 bpm vs 167.6 ± 6.3 bpm; *P* < 0.001, *P*-adjusted for Bonferroni = 0.040 and *P* = 0.001, respectively). There were no CC genotypes in the studied sample. Moreover, a statistically significant difference was found between AA genotypes for the *ACE* gene and the AG + GG with respect to the maximum VE (200 ± 16 L/min vs 162 ± 19 L/min, *P* = 0.040, Figure 2E).

#### HIF1A Genetic Variant Is Associated With Somatotype

When we studied the relationship between the different SNPs and the results of the somatotype, we found a statistically significant association between *HIF1A* genotypes and the mesomorphic component. Precisely, among the CC cyclists, 8.33% presented a low mesomorphic value, 91.7% a moderate value and 0% a high value; while amongst the CT individuals, 33.33% presented a moderate result and the rest 66.7% a high value (*P* = 0.029). There were no TT individuals in the studied sample.

#### Genes Associated With Dietary Records

When analyzing the association of the different genotypes of each SNP and the results of the dietary variables, we found different interesting associations.

**FIGURE 3:**
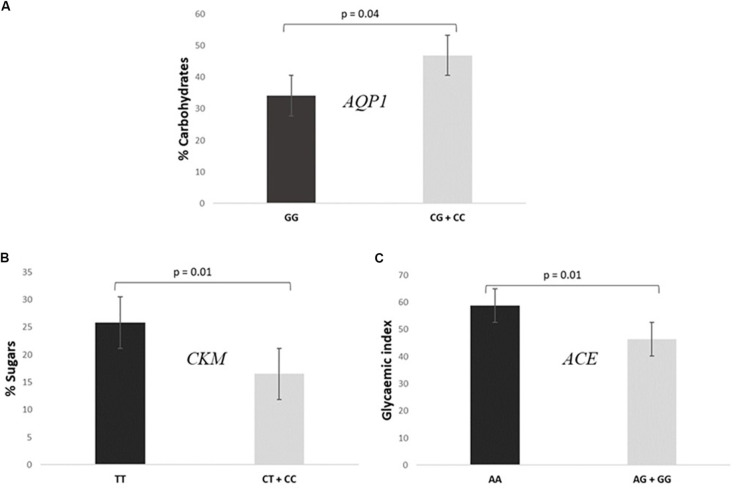
Association of SNPs included in the prediction algorithm GES and specific dietary variables. **(A)** Consumption of% total caloric value (TCV) carbohydrates according to *AQP1* rs1049305. **(B)** Consumption of% TCV sugars according to *CKM* rs8111989. **(C)** Mean glycemic index values according to *ACE* rs4343.
• Participants TT for the *CKM* had a higher consumption of sugars (% TCV) than CC + CT individuals (25.9 ± 9.2% vs 16.5 ± 3.9%, *P* = 0.010, Figure 3B).
• Cyclists AA for the *ACE* presented a higher mean glycemic index consumption than AG + GG (58.9 ± 1.1 vs 46.5 ± 11.7, *P* = 0.010, Figure 3C).

• Subjects GG for the *AQP1* presented a lower intake of carbohydrates [% total caloric value (TCV)] than CG + CC individuals (34.2 ± 11.4% vs 46.9 ± 8.5%, *P* = 0.040), as shown in Figure 3A.

**FIGURE 4:**
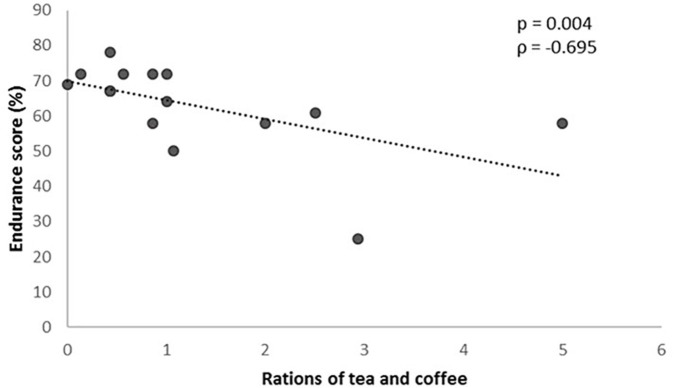
Rations of tea and coffee consumed according to the genetic endurance prediction score (GES).

On the other hand, with regard to the food frequency questionnaire, and inverse correlation between the number of coffee and tea rations consumed per day and the result of the GES was observed (*P* = 0.004), so that the higher the consumption the lower the GES (Figure 4).

## Discussion

The present research provides new information regarding the link between genetics and sport performance from different angles.

The most important result was the potential validation of an algorithm prediction of genetic susceptibility to endurance abilities. At present, cardiovascular exercise test is considered the gold standard assessment of endurance performance (Hausen et al., 2018). We observed that those subjects with a higher value in the GES, presented significantly better results in the cardiovascular exercise test according to VO_2_ in VT1, VO_2_ in VT2 and most importantly, VO_2_ max which is internationally considered the key measure of aerobic capacity (Hausen et al., 2018). Actually, VO_2_ max obtained in this kind of test represents the highest capacity of oxygen consumption during a maximal exercise (Shete et al., 2014). Additionally, VT1 is the point at which the aerobic metabolism is no longer the major energy source and the anaerobic metabolism begins to be used. The exercise intensity at which VT1 occurs is considered the highest submaximal level tolerated by an individual for long time periods (Herdy et al., 2016). Hence, we hypothesized that higher values of VO_2_ at VT1 may predict higher VO_2_ max, which leads to better endurance conditions. Concerning VT2, it is the point where the demand of oxygen by muscles exceeds the mitochondrial supplies and consequently, energy production begins predominantly anaerobic. It begins when lactate starts to accumulate in blood which is usually at 60–70% VO_2_ max (Albouaini et al., 2007). The VO_2_ at VT2 is considered a submaximal index of exercise capacity and endurance performance, so that the higher VO_2_ at VT2, the better endurance results (Coyle et al., 1988; Herdy et al., 2016). These achievements may complement the association found between a genetic score based on a GWAS study and the improvement of the VO_2_ max in a sedentary population carried out by previous researchers (Bouchard et al., 2011).

When we analyzed the association between the different genotypes individually and the cardiovascular test results, we also found some interesting correlations. In this regard, individuals presenting the favorable genotypes for endurance sports according to *AQP1* (Martinez et al., 2009; Saunders et al., 2015), showed better results in the cardiovascular exercise test (higher levels of VO_2_ in VT1 and % of VO_2_ in VT1 in relation to VO_2_ max), than GG homozygous. The physiological explanation for this association may lie in the *AQP1* encoded membrane protein role. The aquaporin 1 water channel is implicated in the transport of water, maintaining the osmotic balance between the blood and the cells (Frigeri et al., 2004). During prolonged exercise, body temperature is regulated by controlling the distribution of body fluid; water passes from the intracellular to the extracellular spaces and evaporates by sweating. An inadequate loss of sweat during exercise, especially in hot climates, will negatively affect athletic performance (Kenny, 2014). Thus, we hypothesize that CC genotype is associated with an efficient state of cellular hydration and body temperature regulation which leads to a better endurance performance.

Moreover, favorable genotypes for endurance performance of the *PPAR*α (Lopez-Leon et al., 2016) presented significantly higher values of VO_2_ in VT2 than CC + CG. This gene regulates the expression of other genes involved in the metabolism of energy, fats and sugars in the skeletal muscle among other tissues (Duval et al., 2004). Given its involvement in these processes, it is hypothesized that it is activated during endurance exercise (Lopez-Leon et al., 2016). Another suggested explanation for the association of *PPAR*α genotypes with endurance performance has to do with the type of fibers in the skeletal muscle. In this sense, in a cohort of 786 Russian athletes it was observed that GG homozygous presented significantly higher percentage of type I muscle fibers than the other genotypes (Ahmetov et al., 2006). These skeletal muscle fibers are classified into two types: type I or slow contraction and type II or rapid contraction fibers. Type I fibers have greater resistance to fatigue and predominate in resistance athletes, while the type II ones are adapted to strong and explosive muscle contractions and predominate in athletes who perform power sports (Cartee et al., 2016).

Regarding the *GABPB1*, the individuals presenting the AA genotype showed a lower maximal HR and lower HR in VT1 and VT2 than AC genotypes. This result is in accordance with other studies that also consider this genotype favorable for endurance sports (Eynon et al., 2013) as it has been described that endurance athletes present lower HR in maximum aerobic traits (Zaniqueli et al., 2014). The *GABPB1* gene encodes the GA-binding protein transcription factor, which is implicated in the regulation of the mitochondrial function generating ATP energy (Dinkova-Kostova and Abramov, 2015) what might explain the implication of this gene in the individual endurance capacity.

Additionally, AA genotypes for the *ACE* gene presented higher maximum VE than the AG + GG genotypes. The AA genotype of the rs4343 is considered equivalent to the II genotype of the *ACE* I/D rs4340 (Glenn et al., 2009) which at the same time is the most studied favorable genotype for endurance performance (Ma et al., 2013). Maximal VE has been reported to be directly correlated with VO_2_ max (Keramidas et al., 2010; Malekmohammad et al., 2012). Accordingly, it is suggested that muscles of endurance athletes require higher values of VO_2_ so that their VE during exercise is higher.

Hypoxia-inducible factor-1 (HIF1) regulates oxygen homeostasis in mammalian cells and in particular, it seems to have a role during high intensity exercise, helping the skeletal muscle to adapt to low oxygen concentrations (Freyssenet, 2007). According to the association between somatotype variables and the different genotypes, individuals carrying the T allele of the *HIF1A* SNP presented moderate or high mesomorphic values, while none of the CC homozygous had a high mesomorphic component. Precisely, allele T is associated with power-oriented athletes (Drozdovska et al., 2013). This makes sense as high mesomorphic individuals are characterized by high skeletal muscle mass, needed for power anaerobic exercises (Gutnik et al., 2015). Besides, as cycling is more an endurance-oriented sport than a power sport, it also makes sense that the mesomorph was the component with the lowest value among the sample.

When we analyzed the association of the different genotypes and the dietary records, we also found diverse significant associations. The cyclists with a favorable genotype for endurance sports regarding the *AQP1* presented a higher intake of carbohydrates which we hypothesize that would probably be due to maintain the glycogen stores needed for long distance exercise (Alghannam et al., 2018). Similarly, individuals with an unfavorable genotype for power sports according to *CKM* consumed higher amounts of sugars (Chen et al., 2017). Commonly, it is considered that genotypes unfavorable for power are favorable for endurance sports and vice versa, which might explain this association. To our knowledge, this is the first time that *AQP1* and *CKM* genotypes have been associated with dietary intake.

Moreover, individuals presenting a favorable genotype for endurance sports according to *ACE* gene presented a higher mean glycemic index intake than the other genotypes. Although it seems clear that carbohydrate consumption is needed to maintain glycogen stores, whether these macronutrients are preferable to be complex or with a high glycemic index appears to be controversial, as there are studies that point out that moderate glycemic index diets improves exercise performance (Durkalec-Michalski et al., 2017) while others support the low glycemic index (Durkalec-Michalski et al., 2018). A possible explanation of this controversy may lie in the fact that the unfavorable *ACE* genotype for endurance performance has also been linked to lower glucose tolerance (Schuler et al., 2017). Thus, we hypothesize that *ACE* AA individuals might take advantage in endurance sports by consuming a moderate glycemic index diet as they can metabolize glucose in a better way. However, we are aware that more studies in this field are needed to confirm this hypothesis.

Finally, an interesting association between the habit of consuming caffeinated beverages and the result of the GES was observed. A possible explanation for this inverse correlation might be that athletes less genetically predisposed to endurance sports where energy is a key factor, are more likely to use ergogenic aids such as caffeine. However, we have to consider that the food frequency questionnaire used gives overage data of the last year, but we do not have data on dietary intakes in specific time frames such as competitions.

## Conclusion

The outcomes of the present study confirm a positive relationship between an endurance prediction algorithm and the results of a cardiopulmonary exercise test. Moreover, *AQP1*, *PPAR*α, *GABPB1*, and *ACE* genes were individually related with endurance performance. Besides, *HIF1A* showed an association with the somatotype and *AQP1*, *CKM*, and *ACE* genes were associated with the athletes’ dietary intake. In addition, an inverse association between the habit of consuming caffeinated beverages and the GES was observed.

This information may facilitate the design of larger studies implicated in the prediction of sports capacities, which may facilitate the finding of genetically talented athletes, improve their training and dietary habits, as well as help in the improvement of physical conditions of amateur athletes.

## Data Availability Statement

The datasets presented in this article are not readily available because are part of the GENYAL Platform for clinical trials in nutrition and health (https://www.food.imdea.org/services/Platform-Clinical-Trials-Nutrition-and-Health) database. This is a database that is currently registered as a collection under the Spanish rules which by policy of the Center will be public afterwards once the data of the entire expected population is gathered. Requests to access the datasets should be directed to ana.ramirez@imdea.org.

## Ethics Statement

The studies involving human participants were reviewed and approved by the Research Ethics Committee of the IMDEA Food Foundation. The patients/participants provided their written informed consent to participate in this study.

## Author Contributions

AR, GR, JS, and VL-K proposed, funded, and designed the research. FL-S, JR-A, and JS did the recruitment of the sample and the monitoring of the cardiopulmonary exercise test. RI, IE-S, EB-R, HM-P, and EA-A performed the anthropometric measurements, body composition analysis, dietary records and, DNA collection and genotyping. GC performed the statistical analysis. RI wrote the first draft of the manuscript. IE-S, FL-S, and JR-A wrote sections of the manuscript. All authors contributed to manuscript revision, read and approved the submitted version.

## Conflict of Interest

The authors declare that the research was conducted in the absence of any commercial or financial relationships that could be construed as a potential conflict of interest.

## Footnotes

1 www.r-project.org
